# *Yokenella regensburgei* urinary tract infection in an immunocompetent patient: a case report

**DOI:** 10.1099/acmi.0.000571.v4

**Published:** 2023-10-17

**Authors:** Annie Sheeba V, Thangamani Suji, Selvin Theodore Jayanth, Rani Diana Sahni

**Affiliations:** ^1^​ Department of Clinical Microbiology, Christian Medical College, Vellore, Tamil Nadu, India; ^2^​ Department of Urology, Christian Medical College, Vellore, Tamil Nadu, India

**Keywords:** AmpC, UTI, *Yokenella*

## Abstract

*
Yokenella regensburgei *, belonging to the order *Enterobacterales *, is a rare and emerging human pathogen reported to cause both superficial and invasive infections. The 13 case reports in the literature worldwide highlight blood, bone and wound infections. To our knowledge this is the first case description of *Y. regensburgei* causing a urinary tract infection in a 69-year-old immunocompetent patient which was isolated in two separate specimens and identified using matrix-assisted laser desorption ionization time-of-flight MS. It was found to be susceptible to most antimicrobials but resistant to penicillin, amoxicillin-clavulanate, cefoxitin and colistin. Inducible chromosomal *ampC* resistance was demonstrated on disc approximation testing, and *bla*YOC-1 class C beta-lactamase, beta lactamase superfamily and MBL fold metallo-hydrolase genes were found on whole genome sequencing.

## Data Summary

The whole genome sequence of the isolate was deposited in the National Center for Biotechnology Information (NCBI) GenBank Short Read Archive under BioProject accession number PRJNA926362.

## Introduction

*Yokenella regensburgei,* a Gram-negative bacillus belonging to the order *Enterobacterales,* is the only recognized species of the genus *Yokenella* [1]. It has been isolated from the intestine of insects, reptiles and well water from the environment. *Y. regensburgei* has been reported to be a rare pathogen causing infection mainly in immunocompromised patients. It phenotypically resembles *Hafnia alvei* and has been isolated worldwide from blood, wounds, bone and joint aspirates in humans [1, 2]. Clinical presentation of a patient with urinary tract infection (UTI) associated with this organism has not been described in the literature although a solitary report mentions isolation from urine [3].

Thus, we present and discuss the first case identified at our centre of *Y. regensbergei* causing a UTI.

## Case description

### Case details

A 69-year-old retired man, with benign prostatic hypertrophy (BPH), had undergone a transurethral resection of the prostate 5 years previously. A year later he developed poor urine flow, straining to void and incomplete voiding. He had developed near urinary retention and UTI multiple times in the previous 2 years. He was diagnosed to have a urethral stricture and had undergone urethral dilatation elsewhere a year previously. In the 2 weeks prior to presentation at our centre, he had complained of increased frequency, burning micturition and dribbling. He did not have fever. On investigation, he was found to have a tight bulbar stricture on cystoscopy and micturating cystourethrogram (MCU) with a high post-void residue. Ultrasonography showed an enlarged prostate with no hydronephrosis. His renal functions were altered with an estimated glomerular filtration rate of 42 ml min^−1^/1.73 m^2^ and serum creatinine was elevated at 1.76 mg%.

He had no other no comorbidities. His fasting glucose was 116 mg dl^−1^ and 2 h post-prandially this was 154 mg dl^−1^. His haemoglobin was 14.8 g% and he was not on any medication. He did not give any history of smoking or alcohol intake. Investigations did not reveal any blood-borne virus.

### Urine analysis, culture and susceptibility

Urine analysis detected elevated white blood cells (WBCs), leukocytes and nitrates (urine routine analysis: WBC: 31 per high-power field, bacteria: 2+, leukocytes: 2+, nitrates: 1+). A concomitant clean mid-stream semi-quantitative urine culture was performed with 10 µl of the specimen on sterile and quality-passed 7 % sheep blood agar and MacConkey agar media, prepared in the Department of Clinical Microbiology. Significant growth indicated by >100 000 c.f.u. ml^−1^ *Y. regensbergei* was isolated in culture at 37 °C under ambient air, and the Gram-stained smears on the specimen were consistent with an inflammatory response. Three days later a suprapubic urine aspirate (SPA) was sent for culture to confirm the findings of mid-stream collection. The same culture findings were obtained on the SPA specimen.

#### Detection

Preliminary screening of the isolate revealed a glucose- and mannitol-fermenting, motile organism, which was lactose- and sucrose non-fermenting, and indole was not produced. Citrate was utilized after 48 h of incubation [MMTPC: ++−/(+)−+]. Final identification was determined by complete biochemical characterization and by matrix-assisted laser desorption ionization time-of-flight (MALDI-TOF) MS (bioMérieux; Version 3.2) with a 99.9 % high-confidence identification.

#### Susceptibility

Susceptibility was determined by Kirby–Bauer disc diffusion methods according to CLSI guidelines [4]. Both isolates were susceptible to nitrofurantoin, co-trimoxazole, aminoglycosides, fluoroquinolones, third- and fourth-generation cephalosporins and beta-lactam inhibitor combinations, and carbapenems. Resistance was noted to penicillin, amoxicillin, ampicillin, amoxicillin-clavulanate, cefoxitin and colistin.

**Fig. 1.**
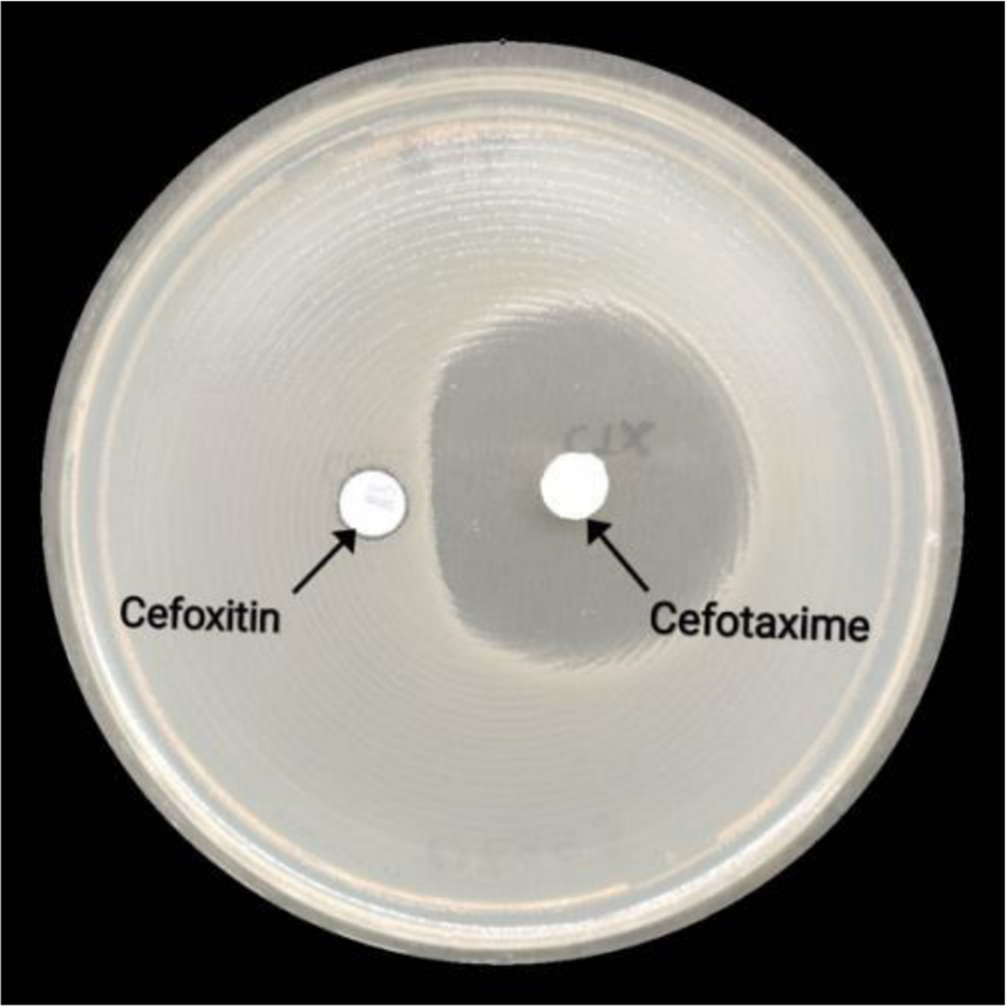
Phenotypic detection of the inducible chromosomal *ampC* gene via a disc approximation test.

Multiplex PCRs to detect plasmid-mediated extended-spectrum beta-lactamase (ESBL) (TEM, SHV, VEB, PER, GES, SPM) and carbapenem resistance genes (KPC, NDM, VIM, IMP, OXA-48like) were negative. Multiplex PCR for the genes CMY/MOX, CIT, DHA, ACC, ACT/MIR and FOX, as described by Pérez-Pérez FJ and Hanson 2002 [5], to determine carriage of chromosomally mediated *ampC* genes, was also negative. Phenotypic detection of a chromosomally encoded *ampC* gene in the presence of a potent inducer of *ampC* (cefoxtin) revealed a positive result as indicated by a flattening of the zone of inhibition around cefotaxime 30 µg when placed close to the inducer 30 µg cefoxitin (disc approximation test) (Fig. 1).

Since PCR did not detect the presence of *ampC* genes, whole genome sequencing was performed using the Ilumina platform and raw reads were assembled using skesa and annotated with PGAP and eggNOG. This revealed blaYOC-1 class C beta-lactamases, beta-lactamase superfamily and MBL fold metallo-hydrolase genes, leading to the beta-lactam resistance as observed phenotypically, while the *arnD*, *arnT* and *pmr*D genes were identified by eggNOG contributing to colistin resistance (BioProject accession number PRJNA926362).

### Outcome

Following the investigations, the patient underwent a suprapubic catheter placement and subsequently was treated with cefaperazone-sulbactam for 5 days (1.5 g intravenously). His symptoms resolved and he was planned for a substitution urethroplasty.

## Discussion

*Y. regensburgei* was initially identified as NIH biogroup 9 by the National Institute of Japan and as enteric group 45 by the Centers for Disease Control and Prevention (CDC). In 1984 it was named as *Y. regensburgei* by Kosako *et al*., while the CDC named it *Koserella trabulsii* replacing enteric group 45. Later, both these organisms were found to represent the same organism and the name *Y. regensburgei* had priority over *K. trabulsii* based on the Bacteriological Code and the CDC approved *Y. regensburgei* in 1991 [1, 4]. It has been usually isolated from insect intestine, reptiles, raw salads and well water, while from humans it has been isolated worldwide, from superficial and invasive site specimens.

**Fig. 2.**
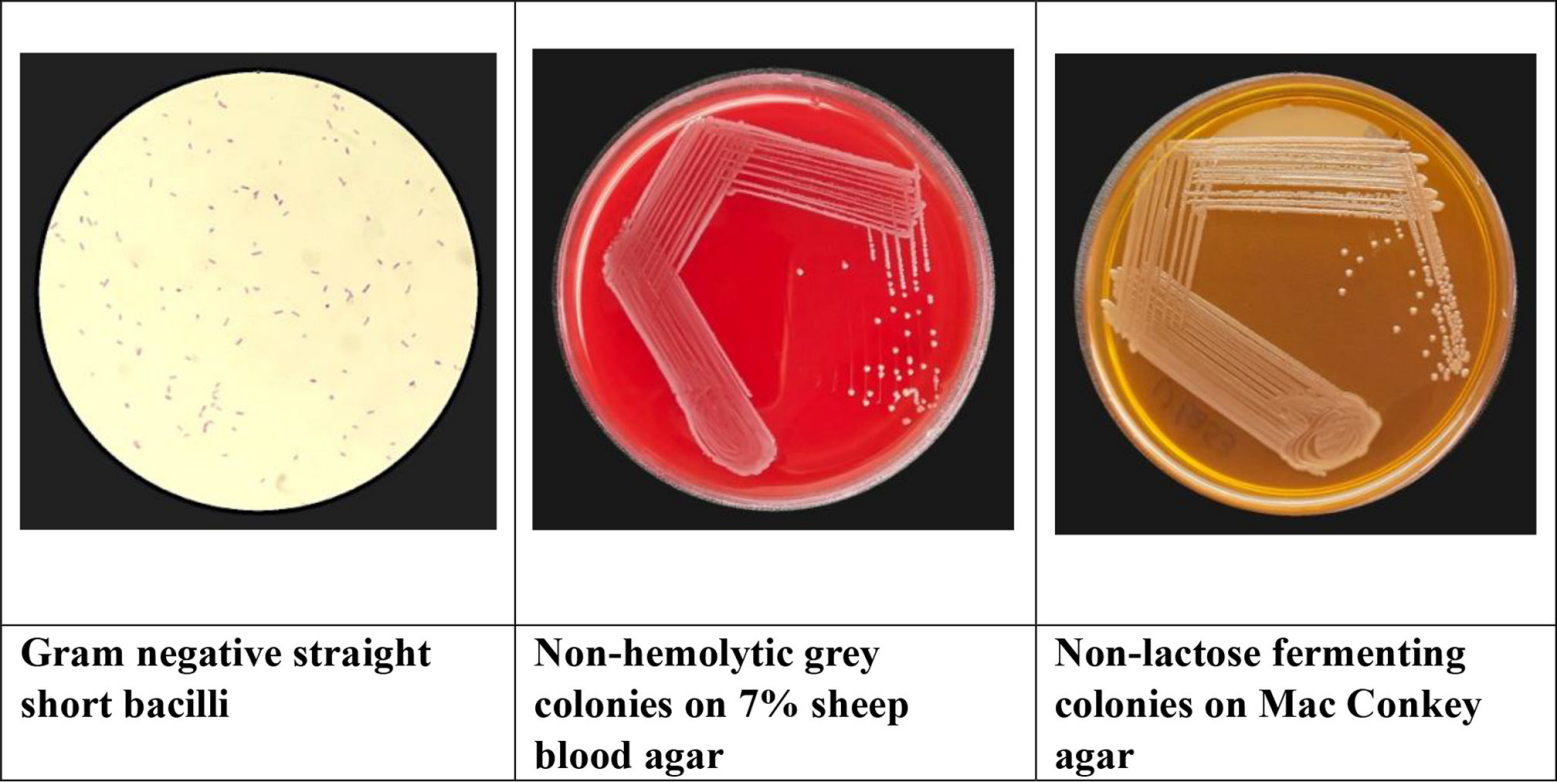
*Yokenella regensbergei*: microscopic appearance and culture description.

*Y. regensburgei* as a clinical pathogen is not well described because of the difficulty in identifying it in low-resource laboratories. It is a nondescript short Gram-negative bacillus and grows as non-haemolytic, non-mucoid grey colonies on sheep blood agar (7 %) and is non-lactose-fermenting on MacConkey agar (Fig. 2).

A wide range of biochemicals or automated identification systems are required to identify the organism as it closely resembles and can be misidentified as *Hafnia alvei, Enterobacter* spp. and *Serratia* spp., due to its biochemical similarity.

Previous case reports regarding *Yokenella* describe a varied presentation, namely septic arthritis and transient bacteraemia (Abbott & Janda, 1994), venous ulcers (Fajardo Olivares et al., 2005), septic shock with abdominal abscess (Fill & Stephens, 2010), cellulitis with septic shock [6], pyrexia resembling enteric fever (Sarika et al, 2013), necrotizing fasciitis (Wright et al, 2019), osteomyelitis (Penagos et al, 2015), otitis media (Gina et al., 2021), finger osteitis (Denes et al., 2021) and osteoarticular infection (Guilarde, 2021). There is no report describing UTIs associated with the bacterium, although Aziz [3] reports its isolation from urine Table 1.

**Table 1.** *
Yokenella regensburgei* previously reported in the published literature

Author	Year	Geographical region	Clinical diagnosis	Underlying condition	Culture source	Laboratory diagnosis
Abbott *et al*.	1994	California	Septic arthritis Transient bacteraemia	Alcohol abuse Alcohol abuse, pancreatitis	Wound sample Blood	Biochemicals
Fajardo *et al*.	2005	Spain	Venous ulcer	Chronic renal insufficiency	Wound	Biochemicals
Fill *et al*.	2010	USA	Septic shock, abdominal abscess, pneumonia	Oesophageal and renal carcinoma, diabetes mellitus	Blood	Biochemicals
Lo *et al*.	2011	Taiwan	Cellulitis with severe sepsis	Diabetes mellitus and was on immunosuppressive drugs	Two blood samples	Biochemicals
Sarika *et al*.	2013	India	PUO	Not mentioned	Blood	Biochemicals, partial 16S rRNA PCR
Bhowmick and Weinstein	2013	USA	Soft tissue disease	Multiple myeloma, autologous stem cell, steroid use	Blood and bulla aspirate	BD Binax, partial 16S rRNA
Penagos *et al*.	2015	Colombia	Osteomyelitis	Invasive pituitary macro-adenoma	Bone	Vitek 2
Aziz	2015	Iraq	Identification of *Yokenella regensburgei* as an agent of urinary tract infection. Occurrence rate of 0.33 %. No case description		Urine	Vitek 2
Xiangbo Chi	2017	China	Septicaemia	Human immunodeficiency virus	Blood	Biochemicals
Wright *et al.*	2019	USA	Necrotizing fasciitis	Orthotopic liver transplantation on immunosuppressents	Blood	Biochemicals, MALDI-TOF
Gina na *et al*.	2021	Korea	Otitis media	Immunocompetent	Pus	MALDI-TOF, 16S rRNA
Denes *et al*.	2021	France	Finger osteitis	Immunocompetent	Tendon sheath infection	MALDI-TOF
Guilarde	2021	Brazil	Osteoarticular infection	Immunosuppressive drugs	Knee joint fluid	Vitek 2

PUO, Pyrexia of unknown origin.

Identification of rare pathogens such as this has been made possible with the help of automated machines such as bioMérieux’s Vitek-2 and MALDI-TOF MS, BD Phoenix or a wide array of biochemicals. Of the 13 available reports, nine investigators used newer automated or molecular identification methods, all these reports coming in the last decade (Table 1). The unique protein pattern of rarer pathogens aids in high-confidence identifications.

For biochemical identification purposes, *Yokenella* is motile, ferments the carbohydrates mannitol, glucose, arabinose, rhamnose, maltose, xylose and trehalose but not lactose, sucrose, dulcitol, adonitol, inositol or sorbitol. It is methyl red test-positive. It produces gas on fermentation and utilizes citrate slowly, which can be detected after 48 h. It also decarboxylates the amino acids lysine and ornithine. As a strong fermenter, *Yokenella* can be differentiated from *H. alvei* by the methyl red test, citrate utilization, and inositol and sorbitol fermentation, from *Enterobacter* spp. by the methyl red test and lysine decarboxylation, from *Serratia* spp. by the methyl red test, malonate, arabinose, gelatin liquefaction and DNAase test [6–8], and in susceptible strains by their characteristic antimicrobial resistance.

A study by Stock *et al*. on 10 strains of *Y. resenburgei* found that, genotypically, all members of the species had the *ampC* gene and strongly expressed inducible β-lactamses. Their study also reported that *Yokenella* species were resistant or had intermediate susceptibility to penicillin G, oxacillin, amoxicillin, amoxicillin-clavulanate, cefalcor, cefoxitin, fusidic acid, linezolid, glycopeptides, rifampicin, fusidic acid, streptogramins and lincosamides but were sensitive to several β-lactams, aminoglycosides, chloramphenicol, cotrimoxazole, fosfomycin and tetracyclines [6, 7]. Our isolate had a similar resistance profile, with a positive disc approximation test detecting inducible *amp*C gene carriage and whole genome sequencing (WGS) identified *bla*YOC-1 class C beta-lactamases.

In our tertiary care centre and high-capacity laboratory, which receives approximately 50 000 urine specimens annually with all organism identifications made with a wide range of biochemicals, VITEK-2 or MALDI-TOF MS or organism-specific PCR, this is the first isolate of *Yokenella* identified, highlighting its rarity. It was isolated in two specimens sent 3 days apart, the second of which was a direct suprapubic aspirate, confirming its presence and pathogenicity in the urinary tract. Further, of the 17 WGS deposits of this organism in the genome database only four were isolated from humans and none have been reported from UTIs, also highlighting its rarity despiteits world-wide pathogenic potential. Finally, our patient, a known case of urethral stricture with recent symptoms of cystitis, was treated with cefaperazone-sulbactam intravenously for 10 days, which is one of the standard treatment options in the management of complicated urology cases in our centre. His symptoms resolved and he was planned for a substitution urethroplasty, which proceeded uneventfully.

## Conclusion

*Y. regensbergei* is a rare pathogen that can invade the urinary tract irrespective of the host’s immune status. It can be identified by both complete conventional biochemicals and automated systems. The key to its identification is its resistance to penicillins, cefoxitin, amoxicillin-clavulanate and colistin. Genotypically, it carries the *amp*C gene chromosomally, and thus third-generation cephalosporins are best avoided in the management of these infections.

To our knowledge, this is the first case description of *Y. regensbergei* causing a UTI.
